# RIPK3-deficient mice were not protected from nephrotoxic nephritis

**DOI:** 10.1186/s12882-018-0850-4

**Published:** 2018-03-14

**Authors:** N. R. Hill, H. T. Cook, C. D. Pusey, R. M. Tarzi

**Affiliations:** 1 Renal and Vascular Inflammation Section, Hammersmith Hospital, 5N4 Commonwealth Building, W12 0NN, London, UK; 2 Centre for Complement and Inflammation Research, Department of Medicine, Imperial College London, W12 0NN, London, UK

**Keywords:** Necroptosis, RIPK3, Glomerulonephritis, Nephrotoxic nephritis

## Abstract

**Background:**

Necrotizing glomerular lesions are a feature of severe glomerulonephritis. Unlike apoptosis, cellular necrosis has the potential to release damage-associated proteins into the microenvironment, thereby potentiating inflammation. Until recently necrosis was thought to be an unregulated cellular response to injury. However, recent evidence suggests that under certain circumstances receptor mediated necrosis occurs in response to death ligand signalling, one form of which is termed necroptosis. RIPK3, a receptor interacting protein, is a limiting step in the intracellular signalling pathway of necroptosis. A non-redundant role for RIPK3 has been implicated in mouse models of renal ischaemia reperfusion injury and toxic renal injury. The aim of this study was to investigate the role of RIPK3 in nephrotoxic nephritis (NTN), a model of immune complex glomerulonephritis in mice.

**Methods:**

We induced NTN in RIPK3−/− and WT mice, comparing histology and renal function in both groups.

**Results:**

There was no improvement in urinary albumin creatinine ratio, serum urea, glomerular thrombosis or glomerular macrophage infiltration in the RIPK3−/− mice compared to WT. There was also no difference in number of apoptotic cells in glomeruli as measured by TUNEL staining between the RIPK3−/− and WT mice.

**Conclusion:**

The data suggests that RIPK3 is not on a critical pathway in the pathogenesis of nephrotoxic nephritis.

## Background

Necroptosis is a regulated pathway to cell death, which shares several upstream signalling elements with apoptosis and can be triggered by different stimuli including TNF, LPS and FasL. Apoptosis is a controlled process of cell disposal resulting in plasma membrane blebbing, limiting the release of immunogenic intracellular proteins into the environment and leading to phagocytosis of apoptotic cells. However, necroptosis leads to plasma membrane rupture and cell death resulting in the potential release of danger associated molecular patterns (DAMPS), inducing the inflammatory process [1, 2]. Necroptosis is defined as death receptor signalling with caspase inhibition dependent on receptor-interacting serine/threonine-protein kinase 1 (RIPK1) and/or receptor-interacting serine/threonine-protein kinase 3 (RIPK3) activation. In addition to necroptosis, RIP3K has also been shown to be involved in cytokine production via the inflammasome. [3] Necroptosis has been blocked by administration of pharmacological inhibitors including necrostatin(s), which inhibit RIPK1, or genetic deletion of proteins involved in the pathway, including RIPK3. RIPK3−/− mice have been previously shown to be viable and fertile, [4] and initially were found to have no phenotype, although more recent data has shown lymphadenopathy in aging RIPK3−/− mice, over one year of age [5]. Necrostatin, which is known to inhibit RIPK1, is not thought to be a specific inhibitor of necroptosis, since it has also been shown to inhibit apoptosis [6]*.* In the presence of active caspase 8 the apoptosis pathway is active and blocks necroptosis. If caspase 8 is inhibited then RIPK1 is activated. RIPK1 ubiquitinylation initiates the assembly of a signalling complex consisting of phosphorylated RIPK1 and RIPK3; further recruitment of mixed lineage kinase domain-like (MLKL) completes the necrosome causing necroptosis [7].

Linkermann et al. 2012, demonstrated that murine ischaemia reperfusion injury (IRI) is mediated by necroptosis rather than apoptosis. zVAD, a caspase inhibitor was used to block apoptosis and necrostatin-1 (nec-1) to block necroptosis in C57BL/6 mice. Mice underwent 30 min of bilateral renal pedicle clipping and were treated with phosphate-buffered saline, nec-1, or zVAD. Treatment of IRI with nec-1 protected against renal failure but blockade of apoptosis by zVAD did not. Thus, necroptosis can be considered as a main contributor to renal injury in IRI and RIPK3 may be a therapeutic target [8]. Linkermann’s group went on to compare IRI in WT and RIPK3 knockout mice. RIPK3 is the essential downstream partner of RIPK1 in necroptosis and determines whether a cell activates caspase 8 and dies by apoptosis [9]. The RIPK3 knockout mice had significantly improved renal function and reduction in kidney injury compared to WT mice, supporting the importance of RIPK3 and necroptosis in IRI [10].

Many triggers of necroptosis have been identified including FasL [2]. FasL is also thought to be an inducer of apoptosis and mediates several effects including inflammatory cell migration and cytokine responses. Nephrotoxic nephritis in mice is a model of immune complex mediated glomerulonephritis (GN) induced by administration of sheep anti-glomerular basement membrane antibody. In the accelerated model, mice are preimmunised with sheep IgG. This results in proliferative glomerulonephritis with macrophage infiltration and glomerular capillary thrombosis with renal impairment and albuminuria. Mice defective in FasL are protected from nephrotoxic nephritis [11]. There are a number of potential contributors to the protection of FasL defective mice from NTN, including reduced generation of pro-inflammatory cytokines, effects on cell death (including apoptosis or necroptosis) and effects on the immune response. However, there is no definitive histological marker of receptor-mediated necroptosis.

Although RIPK3-mediated necroptosis appears to have an important role in toxic/ischaemic insults that lead to necroptotic cell death [12], the role of RIPK3 in an immune-mediated form of glomerular injury had not been investigated. We therefore examined the role of RIPK3 in NTN using RIPK3−/− mice on a C57BL/6 background. We found that RIPK3−/− mice were not protected from NTN induced renal injury compared to WT mice.

## Methods

### Mice

C57BL/6 mice were purchased from Harlan Olac. RIPK3−/− mice on a C57BL/6 background were obtained under a material transfer agreement from Genentech [4]. All procedures were carried out according to the Institutional guidelines for the care and use of experimental animals and the ARRIVE guidelines. Animal studies were approved by the UK Home Office. Mice were genotyped with primers, RIPK3 rev CGCTTTAGAAGCCTTCAGGTTGAC, RIPK3 fwd1 GCCTGCCCATCAGCAACTC and RIPK3 FWD2 CCAGAGGCCACTTGTGTAGCG as previously published [4, 13]. Age and sex matched C57BL/6 controls were used throughout.

### Induction of nephrotoxic nephritis in RIPK3−/− and control mice

Sheep anti-mouse nephrotoxic globulin (NTS) was prepared as described previously [7]. At day − 5 mice were immunized with 0.2 mg of sheep IgG in a 50:50 mix of saline and complete Freund’s adjuvant (Sigma Chemical, St Louis, MO), as previously described [11]. At day 0 they were injected intravenously with 0.2 ml of NTS. Mice were culled on day 8/9 after the induction of NTN. Experimental mice averaged 2 months old. They were monitored clinically and weighed during each experiment, their average weight was 22.8kgs. All mice from any one individual experiment were culled at the same time/day. 57 mice were culled in total, 25 females and 32 males. A power calculation was used to determine the number of mice needed to detect a 25% decrease in proteinuria. Mice were sacrificed under terminal anaesthesia with exsanguination for collection of terminal blood samples and cervical dislocation. Urine, blood and kidneys were collected.

### Histology and immunohistochemistry

All histological quantification was performed after masking the identity of the section, in order to reduce observer bias. Periodic acid-Schiff (PAS) staining allowed analysis of kidney structure. Glomerular thrombosis was scored in 50 glomeruli per kidney section. Thrombosis was scored according to number of glomeruli, per kidney section, displaying thrombosis (each glomerulus was scored on a 0–4 scale, depending on the proportion of the glomerulus affected by thrombosis). Therefore, the maximum score is 200 for 50 glomeruli assessed.

Macrophage infiltration was quantified by staining for CD68 using antibody FA-11 (AbD Serotec). Fifty glomeruli were counted per section and the total averaged to calculate macrophages per glomerular cross section. Kidneys were fixed in periodate-lysine-paraformaldehyde fixative for 4–6 h, transferred to 13% sucrose in PBS and left overnight at 4 °C. Kidneys were subsequently snap frozen in isopentane cooled with liquid nitrogen then immersed in liquid nitrogen prior storage at − 80 °C. Sections were cut to a thickness of 5 μm, incubated with the anti- CD68 antibody and this was detected using the Polink-2 plus HRP rat detection kit (Newmarket Scientific, Newmarket, U.K.).

For immunofluorescence, kidneys were embedded in cryomatrix (Thermo Scientific) and snap frozen in isopentane cooled with liquid nitrogen, before full immersion into liquid nitrogen. Kidneys were stored at − 80 °C. Cryostat sections were cut to a thickness of 5 μm. To quantitate immunoglobulin, sections were stained with fluorescein isothiocyanate-conjugated goat anti-mouse IgG (Fc-specific) and fluorescein isothiocyanate-conjugated monoclonal mouse anti-sheep IgG clone GT34 (Sigma) as previously described [14]. An Olympus BX4 fluorescence microscope (Olympus Optical, London, UK) was used to visualize sections at × 40 magnification. Images of the sections were captured by the image analysis equipment and the total pixel intensity for each glomerulus was measured and averaged for 15 glomeruli per section. An operator blinded to treatment allocation quantitated all sections from a single experiment at the same session on the same settings.

For cell death detection, tissue was stained with an in-situ cell death detection kit, fluorescein (Roche), according to manufacturer’s protocol. The average number of apoptotic bodies per glomerular cross section over the slide was counted in a blinded fashion.

### Renal function and albuminuria

An overnight collection of urine was obtained from mice housed in metabolic cages with continuous access to food and water, starting the day before sacrifice. Serum urea and urine creatinine were measured in the Department of Clinical Chemistry, Hammersmith Hospital, London, UK. Albuminuria was assessed by radial immunodiffusion against a rabbit anti-mouse albumin. Briefly, a gel was prepared containing rabbit anti-mouse albumin (Sigma). Urine samples or mouse albumin standards were loaded into wells in the gel and incubated for 24 h at 4 °C. The gel was washed, dried, and stained with Coomassie blue, and the albumin concentration of the urine was calculated with reference to the standard curve [15].

### Statistics

Statistics and data plots were generated using GraphPad Prism. Groups were compared by Mann–Whitney *U* test. Data are means ± SEM. Results were considered significant if *p* < 0.05.

## Results

### RIPK3−/− mice on a C57BL/6 background did not demonstrate spontaneous renal pathology

Mice aged between 2 and 4 months were used throughout the NTN experiments. Kidney function of 4-month-old unmanipulated RIPK3−/− mice was normal as determined by albuminuria and serum urea in comparison to WT controls. Kidney histology appeared normal with no increase in glomerular macrophage numbers compared to WT C57BL/6 mice (Table 1).

**Table 1:** Renal Functional parameters and macrophage infiltration in C57BL/6 control and mice following the induction of NTN. Groups were compared by Mann–Whitney *U* test. Data are means ± SEM

	Control mice		NTN mice	
	WT (*n* = 5)	RIPK3−/−(*n* = 5)	WT (*n* = 27)	RIPK3−/− (*n* = 20)
Urinary albumin/creatinine ratio (g/mmol)	0.019 ± 0.006	0.013 ± 0.044	2.1 ± 0.51	2.0 ± 0.74
Serum urea (mmol/l)	7.7 ± 0.84	7.7 ± 0.65	11.1 ± 2.7	21.2 ± 7.2
Macrophages/gcs	1.36 ± 0.21	1.01 ± 0.15	1.9 ± 0.3	2.3 ± 0.2

*Abbreviations*: *gcs* glomerular cross section, *IRI* ischaemia reperfusion injury, *WT* wild type, *RIPK3* receptor interacting protein kinase 3, *NTN* nephrotoxic nephritis

### RIPK3−/− mice were not protected from NTN compared to WT mice

**Fig. 1:**
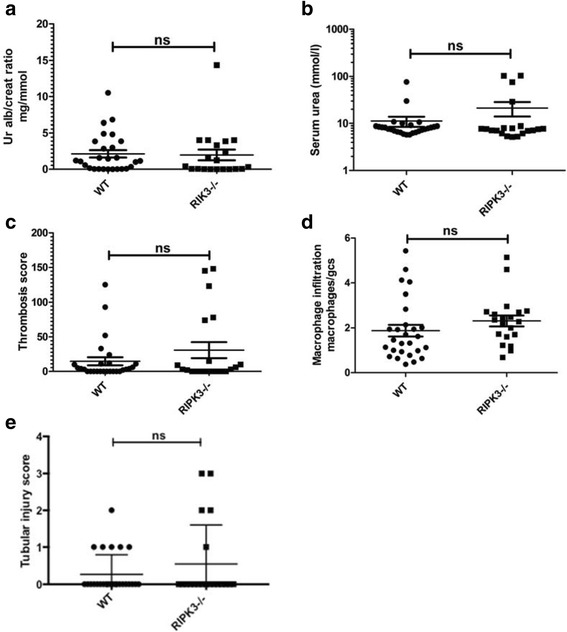
Functional and histological parameters in WT and RIPK3−/− mice with NTN. (**a**) Urinary albumin/creatinine ratio (g/mmol), (**b**) Serum urea (mmol/l), (**c**) Glomerular thrombosis score (maximum score 200), (**d**) Macrophage infiltration (macrophages/glomerular cross section) (**e**) Tubular injury. Groups were compared by Mann–Whitney *U* test. Data are means ± SEM

NTN was induced in RIPK3−/− and WT controls. Experiments were repeated three times. Mice from Experiments 1 and 3 were culled at day 9 and from Experiment 2 were culled at day 8 after the induction of disease. Numbers of mice per group were as follows; Experiment 1, RIPK3−/− = 6, WT = 9; Experiment 2, RIPK3−/− = 7, WT = 8; Experiment 3, RIPK3−/− = 7, WT = 10. In order to increase the statistical power, pooled data from the three experiments were assessed. There were no significant differences between WT and RIPK3−/− in each experiment. There were no significant difference in urinary albumin: creatinine ratio between the pooled RIPK3−/− and WT groups: RIPK3−/− (2.0 ± 0.74 g/mmol *N* = 20) vs WT (2.1 ± 0.51 g/mmol *N* = 27); nor in serum urea: RIPK3−/− (21.2 ± 7.2 mmol/l N = 20) and WT (11.1 ± 2.7 mmol/l N = 27) (Table 1) (Fig. 1a, b). There was no significant difference in the glomerular thrombosis score: RIPK3−/− (72.3 ± 25.7) vs WT 42.8 ± 15.9) or macrophage infiltration: RIPK3−/− (2.8 ± 0.6 macrophages/glomerular cross section) vs WT (3.1 ± 0.6/gcs) (Table 1) (Figs. 1c, d and Fig. 3).

**Fig. 2:**
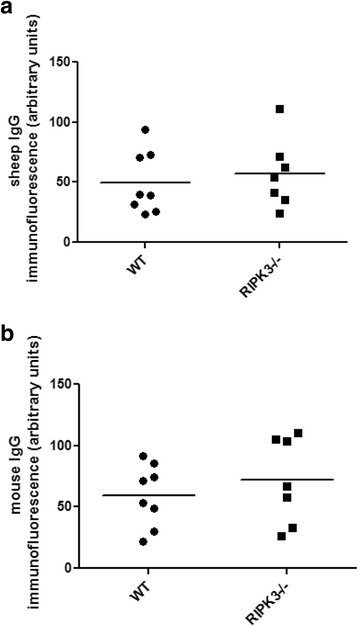
Deposited IgG in WT and RIPK3−/− mice after the induction of NTN. **a**) Deposited glomerular sheep IgG at day 8 after induction of NTN (arbitrary units). **b**) Deposited glomerular mouse IgG on day 8 after induction of NTN (arbitrary units). These are representative data from Experiment 2. Groups were compared by Mann–Whitney *U* test. Data are means ± SEM

**Fig. 3:**
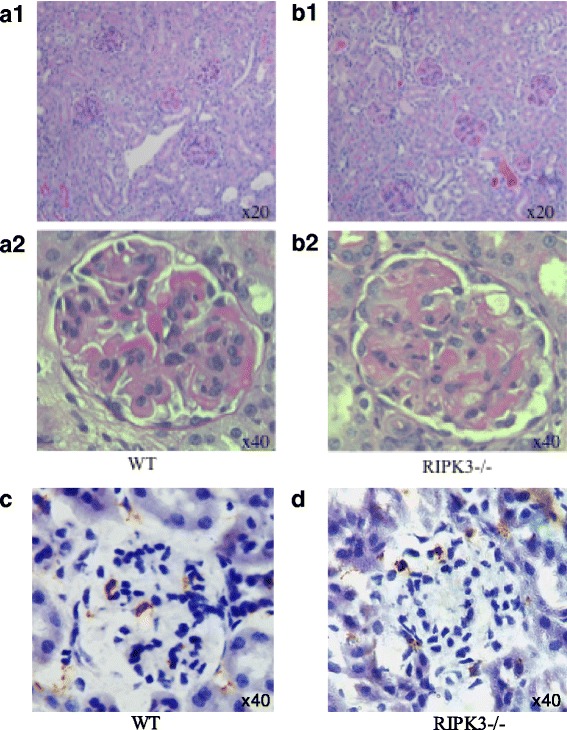
Histology at day 8 after NTN induction. Representative histology from PAS stained sections from Experiment 2, of WT and RIPK3−/− kidney. **a**) WT **b**) RIPK3- /−. Immunoperoxidase staining for CD68 in WT and RIPK3−/− kidney showing glomerular macrophage infiltration following the induction of NTN. **c**) WT **d**) RIPK3−/−

As has been noted by a number of groups previously, including our own, there was variability in the incidence and severity of nephritis between the mice in the groups (Fig. 1). This is not fully explained, but may be related to variability in the immune response of the animals (Fig. 2). Overall, 11/20 (55%), and 18/27 (66%) of animals in the RIPK3−/− and WT groups respectively developed levels of albuminuria significantly higher than normal unmanipulated mice (0.3 g albuminuria/mmol creatinine or more). Similarly 12/20 (60%) and 16/27 (67%) in the RIPK3−/− and WT mice respectively developed glomerular thrombosis higher than unmanipulated mice (thrombosis score of 0), showing varying degrees of histological renal injury, evidenced by glomerular thrombosis and macrophage infiltration (Fig. 1c, d and Fig. 3). Those mice with severe albuminuria also demonstrated severe renal injury and macrophage infiltration across both of the groups. Some animals in both groups developed more severe renal injury, including more severe renal dysfunction and histologial injury including glomerular thrombosis and acute kidney injury (Fig. 1b, c, d and Fig. 3). Unlike other authors [16] we did not see neutrophil infiltration in the interstitium. The data is not consistent with substantial protection of RIP3K−/− mice from NTN.

### Antibody responses were similar in WT and RIPK3−/− mice

Glomerular sheep IgG deposition and mouse IgG deposition were quantified, and showed no significant difference between WT and RIPK3−/− mice (Fig. 2a, b). Results from experiment 2 are representative of all experiments. This was also true for circulating anti -sheep antibody, measured by ELISA according to a previously described method (data not shown) [11].

### Apoptosis was present in WT and RIPK3−/− NTN induced mice with NTN

**Fig. 4:**
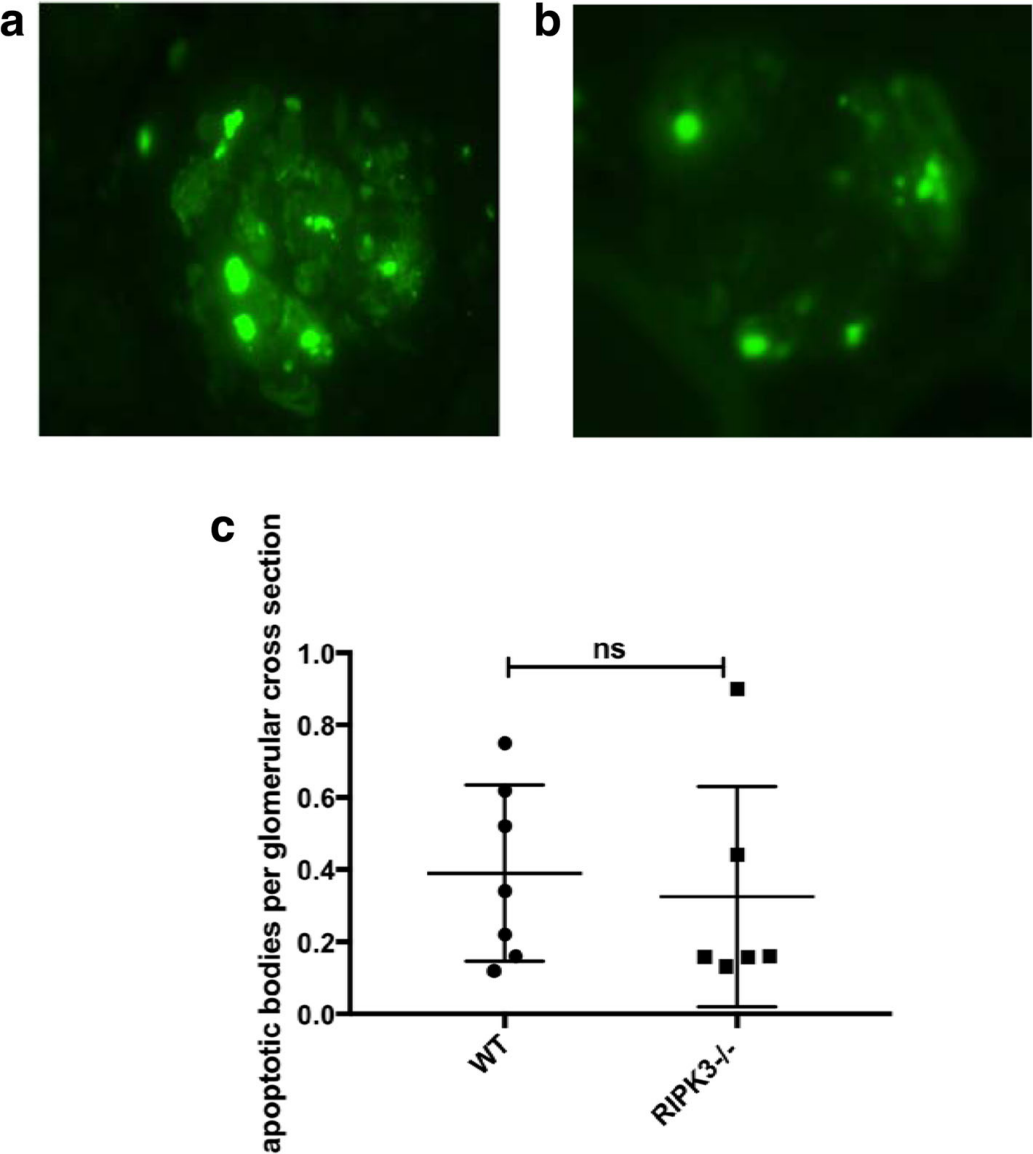
Immunofluorescence detection of apoptotic bodies. Quantification of TUNEL staining, to detect apoptotic bodies in the glomeruli.following the induction of NTN. TUNEL staining from Experiment 2. Fluorescent staining of glomerulus in **a**) WT and **b**) RIPK3−/− mice, highlighting glomeruli from both groups, with higher than average numbers of TUNEL positive cells. **c**) Graph showing mean number of apoptotic bodies per glomerulus for each mouse. Groups were compared by Mann–Whitney *U* test. Data are means ± SEM

In order to ascertain whether cellular apoptosis was impacted by deficiency of RIP3K, apoptosis was analysed in glomeruli of WT (*n* = 9) and RIPK3−/− (*n* = 7) mice from Experiment 2. There was no difference in the number of apoptotic bodies per glomerular cross section between WT and RIPK3−/− WT (0.4±0.1) and RIPK3−/−(0.5±0.1) apoptotic bodies per glomerulus (Fig. 4).

## Discussion

Our data demonstrate that RIPK3 is not on a critical pathway in the pathogenesis of nephrotoxic nephritis, as mice deficient in RIPK3 were not protected from NTN. This result was surprising, since we had previously shown that NTN was dependent on FasL, which triggers signalling via RIPK3, amongst other pathways. Unfortunately there is no specific histologial marker of necroptosis, however there was no evidence of a reduction in renal injury in this model. It is possible that necroptosis occurs in NTN, but that other pathways of disease pathogenesis exist that compensate in the absence of this kinase.

It has been shown that RIPK3 is important in other forms of renal injury, as RIPK3−/− mice were protected from ischaemia reperfusion injury and tubular necrosis [10]. These models are characterized by tubular rather than glomerular injury, suggesting that necroptosis may be more important in tubular than glomerular injury. However, there was no evidence of a trend to protection from tubular damage in the NTN model in our experiments. In addition, recent data would also suggest that RIP3K is not required for folic acid-induced acute kidney injury, which is also more of a tubular form of injury. Therefore, protection of RIPK3−/− mice from renal injury in murine models seems to be context and model dependent [17]. In an immune-mediated model such as the NTN model, the role of cytokines, delayed type hypersensitivity, ADCC mediated by Fc receptors and complement, and migration of neutrophils and macrophages to the inflammatory site, may be more important in glomerulonephritis than in a purely toxic/ischaemic insult. The two best described pathways of regulated necrosis are the RIPK3 dependent pathway involving formation of the necrosome, and the MPT pathway, involving CyD opening of the MPT pore, but several other pathways are emerging [18]. Two independent pathways of necroptosis mediate ischemia-reperfusion injury. IRI was induced in RIPK3−/−, CyD−/−, and double−/− (d−/− mice). In the single knockout mice there was a significant reduction in kidney injury, but with strongest protection in the d−/− mice, suggesting that both pathways are important in the pathology of disease. The RIPK3 pathway of necroptosis does not seem to be important in the mouse model of GN; however it would be of interest to explore other programmed necrosis pathways such as the MPT pathway in this model, as well as assessing pharmacological inhibition of RIPK1, and genetic deletion of MLK.

There are limitations to this study. Firstly, the incidence of renal disease in both groups of mice was somewhat lower than expected. Nevertheless, the findings were consistent throughout all three experiments, and in addition, a similar proportion of mice in each group developed proteinuria and evidence of histological renal injury. Furthermore, some mice in both groups developed more severe renal injury associated with increased urea, glomerular thrombosis and tubular injury, which is not consistent with a significant protective effect of RIPK3 in this model. Data from another group of investigators has recently been published, also demonstrating no protection of RIPK3−/− mice from NTN compared to WT controls, consistent with the data reported in this manuscript [19].

There is no histological marker of necroptosis, and therefore it is not possible to quantitate the cell death of interest in the tissues histologically. TUNEL staining does not clearly differentiate the different forms of regulated necrosis and apoptosis. However, as there was no difference between the RIPK3−/− and WT groups, it would suggest that forms of regulated cell death other than RIPK3-dependent necroptosis compensate for the lack of RIPK3. RIPK3 has also been implicated in the production of pro-inflammatory cytokines via the inflammasome [20]. However, as no effect on disease induction was seen, cytokine levels were not investigated further.

## Conclusion

In conclusion, RIPK3, a key kinase in the pathway of necroptosis, is redundant in the development of NTN, as mice deficient in RIPK3 were not protected from the disease. This is relevant, as RIPK3 has previously been proven to contribute to IRI. It is possible that RIPK3 plays a role in NTN, but that other pathways of disease pathogenesis exist that compensate in the absence of RIPK3.

## Abbreviations

DAMPS: Damage-associated molecular patterns
ELISA: Enzyme linked immunosorbent assay
FASL: Fas ligand
GN: Glomerulonephritis
IRI: Ischemia reperfusion injury
LPS: Lipopolysaccharide
MLKL: Mixed lineage kinase domain like pseudokinase
MPT: Mitochondrial permeability transition
NTN: Nephrotoxic nephritis
NTS: Nephrotoxic serum
PAS: Periodic acid-schiff
PBS: Phosphate buffered saline
RIPK3: Receptor-interacting serine/threonine-protein kinase 3
TNF: Tumour necrosis factor
TUNEL: Terminal deoxynucleotidyl transferase dUTP nick end labelling
WT: Wild type
